# High *ABCC2* and Low *ABCG2* Gene Expression Are Early Events in the Colorectal Adenoma-Carcinoma Sequence

**DOI:** 10.1371/journal.pone.0119255

**Published:** 2015-03-20

**Authors:** Vibeke Andersen, Lotte K Vogel, Tine Iskov Kopp, Mona Sæbø, Annika W. Nonboe, Julian Hamfjord, Elin H. Kure, Ulla Vogel

**Affiliations:** 1 Organ Center, Hospital of Southern Jutland, Aabenraa, Denmark; 2 Institute of Regional Health Research, University of Southern Denmark, Odense, Denmark; 3 Medical Department, Regional Hospital Viborg, Viborg, Denmark; 4 Department of Cellular and Molecular Medicine, University of Copenhagen, Copenhagen, Denmark; 5 National Food Institute, Technical University of Denmark, Søborg, Denmark; 6 Telemark University College, Faculty of Arts and Sciences, Department of Environmental and Health Studies, Telemark, Norway; 7 Department of Genetics, Institute for Cancer Research, Oslo University Hospital, Oslo, Norway; 8 National Research Centre for the Working Environment, Copenhagen, Denmark; University of Colorado Denver, UNITED STATES

## Abstract

Development of colorectal cancer (CRC) may result from a dysfunctional interplay between diet, gut microbes and the immune system. The ABC transport proteins ABCB1 (P-glycoprotein, Multidrug resistance protein 1, MDR1), ABCC2 (MRP2) and ABCG2 (BCRP) are involved in transport of various compounds across the epithelial barrier. Low mRNA level of *ABCB1* has previously been identified as an early event in colorectal carcinogenesis (Andersen et al., PLoS One. 2013 Aug 19;8(8):e72119).

*ABCC2* and *ABCG2* mRNA levels were assessed in intestinal tissue from 122 CRC cases, 106 adenoma cases (12 with severe dysplasia, 94 with mild-moderate dysplasia) and from 18 controls with normal endoscopy.

We found significantly higher level of *ABCC2* in adenomas with mild to moderate dysplasia and carcinoma tissue compared to the levels in unaffected tissue from the same individual (P = 0.037, P = 0.037, and P<0.0001) and in carcinoma and distant unaffected tissue from CRC cases compared to the level in the healthy individuals (P = 0.0046 and P = 0.036). Furthermore, *ABCG2* mRNA levels were significantly lower in adenomas and carcinomas compared to the level in unaffected tissue from the same individuals and compared to tissue from healthy individuals (P<0.0001 for all). The level of *ABCB2* in adjacent normal tissue was significantly higher than in tissue from healthy individuals (P = 0.011).

In conclusion, this study found that *ABCC2* and *ABCG2* expression levels were altered already in mild/moderate dysplasia in carcinogenesis suggesting that these ABC transporters are involved in the early steps of carcinogenesis as previously reported for *ABCB1*. These results suggest that dysfunctional transport across the epithelial barrier may contribute to colorectal carcinogenesis.

## Introduction

Colorectal cancer (CRC) constitutes a major health problem. It is the third most common cancer in the world and the number of cases has been estimated to rise from 1.36 million cases in 2012 to 2.4 million cases in 2035 worldwide [1]. The World Cancer Research Fund estimated that half of the cases may be prevented by dietary and other lifestyle changes [1]. Early detection leading to early treatment will save lives [2]. Therefore, strategies for prevention, early detection and treatment modalities are highly warranted.

CRC may be promoted by a dysfunctional interplay between diet, gut microbes and intestinal immune system via intestinal inflammation [3]. Intestinal inflammation fosters signalling molecules and genotoxic substances introducing genetic and epigenetic changes which may result in carcinogenesis [4]. The ABC transporters are involved in transport of various compounds across the epithelial barrier [5]. Furthermore, the ABC transporters can interact with bacterial pathogens and thereby potentially affect carcinogenesis [6].

The precise mechanisms by which ABC transporters may affect colorectal carcinogenesis are not known, but published studies point at two main mechanisms.

The ABC transport proteins ABCB1 (P-glycoprotein, Multidrug resistance protein 1, MDR1), ABCC2 (MRP2) and ABCG2 (BCRP) are abundant in the gut [7] and localise to the apical plasma membrane of the enterocytes where they export various substrates from the intestinal cells into the intestinal lumen. Thus, by extruding carcinogenic substrates from the intestinal cells, they reduce the absorption of these substrates from the diet, thereby restricting intestinal and systemic exposure. ABC transporters have been found to transport a variety of both harmful and beneficial substrates such as flavonoids and phytoestrogens from fruits and vegetables, short chain fatty acids derived from bacterial degradation of dietary fibres, cooking carcinogens derived from cooking food at high temperature, dietary fatty acids giving rise to pro- and anti-inflammatory signalling molecules, and other food constituents with potential effects on carcinogenesis [8–15].

Furthermore, mice lacking the *ABCB1* gene (abcb1, mdr1a-/-) develop inflammation-related colorectal cancer when exposed to commensal gut microbes [16,17]. Importantly, inflammation was preceded by changes in the tight junctions [18] leading to increased epithelium permeability and activation of pathogen recognition system and changes in the gut microbial composition [18–20] which together promoted inflammation. The inflammation did not develop when the animals were kept in sterile conditions and could be prevented by antibiotics, indicating that bacteria played an essential role [17]. These findings suggest that ABC proteins may be involved in the interaction between luminal antigens and host intestinal immune response. Dietary fat is also able to directly stimulate the pathogen recognition receptors toll like receptors (TLR) [21], thereby activating signalling pathways resulting in interferon-γ (INF-γ) and interleukin (IL)-1β secretion [22] which promote carcinogenesis [3,23,24]. ABC transporters are involved in excretion of signalling molecules such as eicosanoids, INF-γ, tumour necrosis factor-α (TNF-α) and ILs, including IL-1β, thus providing a possible link between ABC transporters, luminal antigens and carcinogenesis [25,26]. In contrast, ABC transporters are probably not involved in the excretion of IL-2, which has a signal sequence [25–27]. The functions of the ABC transporters, including ABCG2, may be related to their transport of phospholipids, thereby generating the cell membrane lipid asymmetry necessary for processes such as vesicle transport and budding, autophagy, and apoptosis [10,28].

We have previously assessed the role of *ABCB1* in colorectal carcinogenesis and the results suggest that low level of *ABCB1* is an early event in colorectal carcinogenesis [29,30]. In the present study our aim was to assess the roles of *ABCC2* and *ABCG2* in the normal-adenoma-carcinoma sequence and we therefore measured *ABCC2* and *ABCG2* mRNA levels in intestinal tissues from patients with colorectal adenomas and CRC. To characterize the role of these ABC transporters in carcinogenesis we measured the mRNA levels in adenoma and carcinoma tissues and morphologically normal tissue from the patients, and in intestinal tissue from healthy control subjects.

## Materials and Methods

### Study cohort

The KAM (Kolorektalkreft, arv og miljø) cohort is based on the screening group of the Norwegian Colorectal Cancer Prevention study in Telemark, and enriched by a series of clinical CRC cases operated at Telemark Hospital (Skien) and Oslo University Hospital (Oslo) as described previously [30–33]. In short, 20,780 healthy men and women, 50–64 years of age, drawn at random from the population registry in Oslo (urban) and the county of Telemark (mixed urban and rural) were invited to have a flexible sigmoidoscopy screening examination. The study was performed in accordance with the Helsinki Declaration. The Regional Ethics Committee and the Data Inspectorate approved the KAM study (S-98190, 2009/2021). The ID number for the study is NCT00119912 at ClinicalTrials.gov. All participants gave verbal and written informed consent. The cohort has previously been analysed [30,32,34–42].

### Biological Material

The present study included intestinal tissue from adenocarcinoma (122) and adenoma (106) cases (94 with mild/moderate dysplasia and 12 with severe dysplasia) and from healthy control cases (18), the latter were defined by having an endoscopy with normal findings. Thus, the 18 samples from healthy controls were sampled throughout the colon. From the adenoma cases, samples were taken from the adenoma and, in addition, from unaffected sigmoideum 30 cm from anus. For the colorectal adenocarcinoma cases, sampling was performed from the surgical specimens. Tissue samples were taken from the cancer and two morphologically unaffected sites adjacent and distant to the cancer (labelled adjacent and distant unaffected tissue respectively). The histology was examined independently by two histopathologists, who categorised the degree of dysplasia as either mild/moderate or severe. Consensus was reached in all cases. Carcinomas were classified according to Dukes’ staging. Tissues were handled immediately in liquid nitrogen after sampling.

### Real-time reverse transcriptase polymerase chain reaction and genotyping

RNA was extracted as previously described [37]. In short, E.Z.N.A, Total RNA Kit II, RNase Free DNase kit I was used according to the instructions by the manufactures and cDNA synthesis was performed using the High-Capacity cDNA Archive Kit (Life Technologies). Messenger RNA levels were normalized to 18S. Since ribosomal RNA constitutes the vast majority of RNA in cells, this corresponds in normalizing to total RNA. We have previously shown in lymphocytes from human volunteers mRNA that levels could be normalized to 18S or β-actin yielding the same result [43].

Quantitative real time RT-PCR for *ABCB1* was performed on a ViiA7 sequence detection system (Life Technologies) in Universal PCR Master Mix (part.no. 4326614, Life Technologies) using pre-developed assays for *ABCC2* (Assay ID: Hs00166123_m1), *ABCG2*: (Assay ID: Hs01053790_m1), β-actin (part.no. 4310881E) and 18S (part.no. 4310893E), (Life Technologies). In a validation experiment, a dilution series was assayed by the comparative C_t_ method (15). The *ABCC2* and *ABCG2* assays were quantitative over a range of 16-fold and 528-fold dilutions, respectively. *ABCC2*, *ABCG2*, β-actin and *18S* were amplified in independent wells in triplicates. The standard deviation of triplicates was 6% or less. The standard deviations on repeated measurements of the same sample (the control) in separate experiments were 22% and 25%, respectively, for *ABCC2* and *ABCG2*, indicating the day-to-day variation of the assays. Negative controls (where the RNA was not converted into cDNA) and positive controls were included in all runs. Samples for which the *18S* values fell outside the limits of the standard curve were excluded from the study. As control, a subset of samples were reanalysed normalised to β-actin.*NFKB1–94ins/del* (rs28362491) genotyping data was retrieved from Andersen *et al*. [30].

### Statistics

All statistical analyses of mRNA levels were performed using SAS (release 9.3, SAS Institute, Cary, NC). Linear regression (PROC GLM) was used to compare mRNA levels in tissue from healthy participants versus tissues from affected participants with adjustment for age and gender. Number of cases with available data on *ABCC2* and *ABCG2* mRNA levels were; controls (16), adenomas (67 with mild/moderate dysplasia, 11 with severe dysplasia, and CRC cases (100 and 98 for ABCC2 and ABCG2, respectively).

A paired t-test (PROC TTEST) was used to compare mRNA levels from unaffected and affected tissue from the same individual. Data on matching samples were available from 66 cases with mild-moderate dysplasia and 11 cases with severe dysplasia. The number of cases for comparison of cancer tissue with distant unaffected tissue and adjacent unaffected tissue were for CRC; *ABCC2*; 63 and 66 cases, *ABCG2*; 82 and 83 cases, respectively.

All values of mRNA expression levels were log-transformed to correct for left-skewed distribution.

Kruskal Wallis and Dunns Multiple Comparison test was used to analyze the association between *NFKB1*–94ins/del (rs28362491) genotypes and *ABCC2* and *ABCG2* mRNA levels using GraphPad Prism 5.

## Results

The study participants are described in Table 1. Adenoma cases and controls had similar age whereas the adenocarcinoma cases were older. The gender distribution differed between controls, adenoma and adenocarcinoma cases. Consequently, all analyses were adjusted for age and gender. There was no correlation between the *ABCC2* and *ABCG2* mRNA levels and Duke’s stage of the carcinoma, age or gender. As a control, we re-determined *ABCG2* and *ABCC2* mRNA levels normalized to β-actin instead of 18S in an independent analysis for a subset of the samples. When the mRNA levels normalized to β-actin was plotted against mRNA levels normalized to 18S, a correlation coefficient, r of 0.91 was found (S1 Fig), indicating a high degree of correlation between mRNA levels normalized to the different reference genes.

**Table 1:** Study participant description.

	Healthy individuals	Adenoma cases		Carcinoma cases	P-values
		Mild/moderate dysplasia	Severe dysplasia		
No. of subjects (N)	18	94	12	122	
Male (N (%))	6 (33)	66 (70)	6 (50)	67 (55)	0.011^a^
Female (N (%))	12 (67)	28 (30)	6 (50)	55 (45)	
Age Mean (SD)	56.8 (4.5)	57.0 (3.6)	55.1 (3.1)	69.5 (11.9)	<0.0001^b^

^a^ P-value for comparison of the distribution of gender in the different groups.

^b^ P-value for comparison of age in the different groups.

### *ABCC2* mRNA levels in intestinal tissue

**Fig 1:**
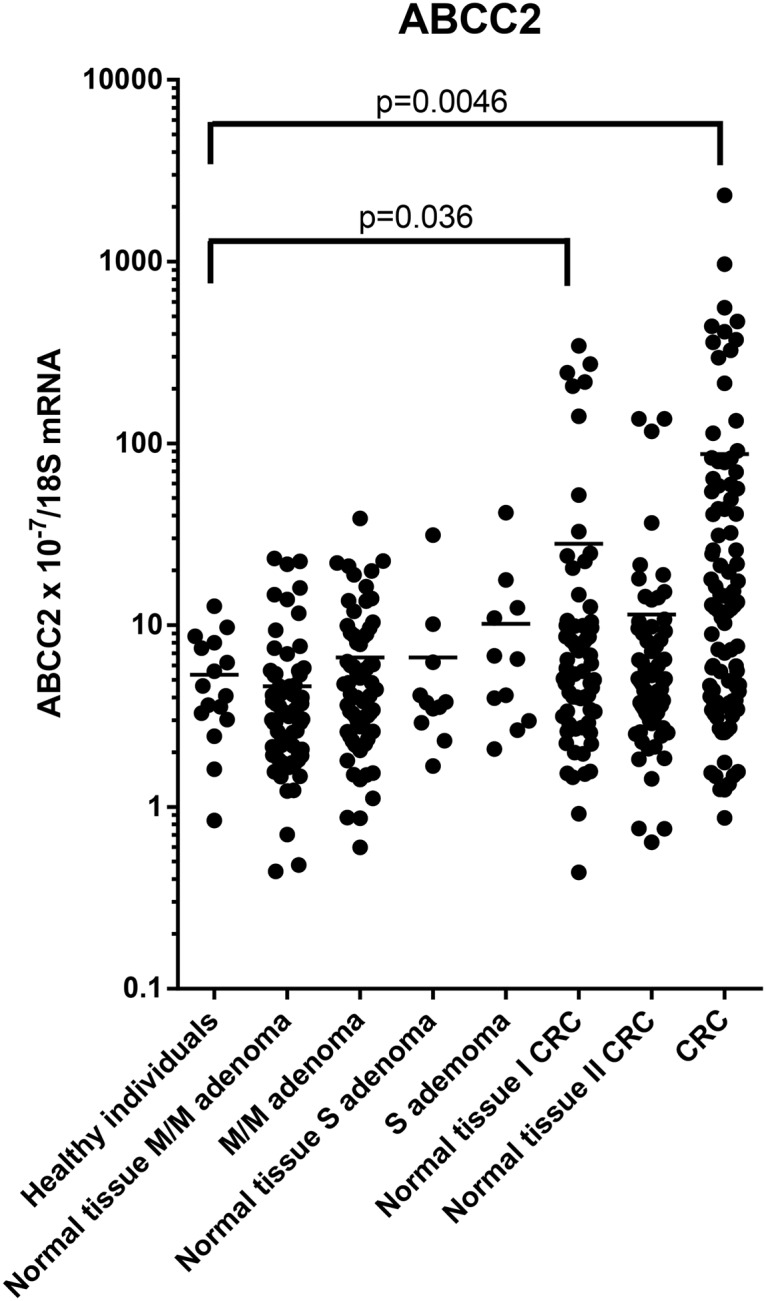
*ABCC2* mRNA levels in morphologically normal and affected tissues normalised to 18S RNA levels. P-values for comparison of the expression levels to the level in healthy controls adjusted for age and gender are indicated. M/M, mild-to moderate dysplasia; S, severe dysplasia; Normal tissue I, morphologically normal distant tissue; Normal tissue II, morphologically normal adjacent tissue. Horizontal bars indicate mean. mRNA expression levels were log-transformed.

The *ABCC2* mRNA levels in the intestinal tissue from the healthy controls, adenoma and CRC cases are shown in Table 2 and Fig. 1.

**Table 2:** *ABCC2* mRNA levels x 10^7^ in intestinal tissues normalised to 18S RNA level.

	unaffected tissue		adenomas/carcinomas		
**Variable**	Mean ± S.D.	P^a^	Mean ± S.D.	P^a^	P^b^
**Healthy individuals**	5.35 ± 3.24				
**Mild moderate dysplasia cases**	4.62 ± 4.79	0.081	6.68 ± 6.77	0.87	0.037
**Severe dysplasia cases**	6.66 ± 8.47	0.88	10.18 ± 11.52	0.27	0.24
**Cancer patients**	28.06 ± 68.84 (distant)	0.036	87.50 ± 270.21	0.0046	0.0037
	11.44 ± 25.58 (adjacent)	0.69			<0.0001

^a^ P-values for comparison of the expression levels to the level in controls adjusted for age and gender.

^b^ P value for the comparison of the expression levels in the morphologically affected and unaffected tissues from the same individual using Paired Student’s t-test. Matching samples were available from 66 cases with mild-moderate dysplasia, 11 cases with severe dysplasia, and 63 and 66 CRC cases (distant unaffected tissue and adjacent unaffected tissue, respectively).

For adenoma cases, *ABCC2* mRNA levels were statistically significantly increased (p = 0.037) in mild and moderate dysplasia as compared to unaffected tissue from the same individual. *ABCC2* mRNA levels were not statistically different from mRNA level in the healthy controls with normal endoscopic findings.

For the CRC cases, the *ABCC2* mRNA levels were statistically significantly increased in cancer tissue compared to levels in both morphologically unaffected distant tissue (p = 0.0037) and unaffected adjacent tissue (p< 0.0001) from the same individual, and compared to levels in tissue from healthy individual (p = 0.0046). *ABCC2* mRNA levels in morphologically unaffected tissue from cancer patients was increased compared to levels in tissue from healthy individuals, but considerable variation in the mRNA levels was seen (Fig. 1) and only the *ABCC2* mRNA level in distant normal tissue was statistically significantly different from the level in tissue from healthy individuals (p = 0.036).

### *ABCG2* mRNA levels in intestinal tissue

**Fig 2:**
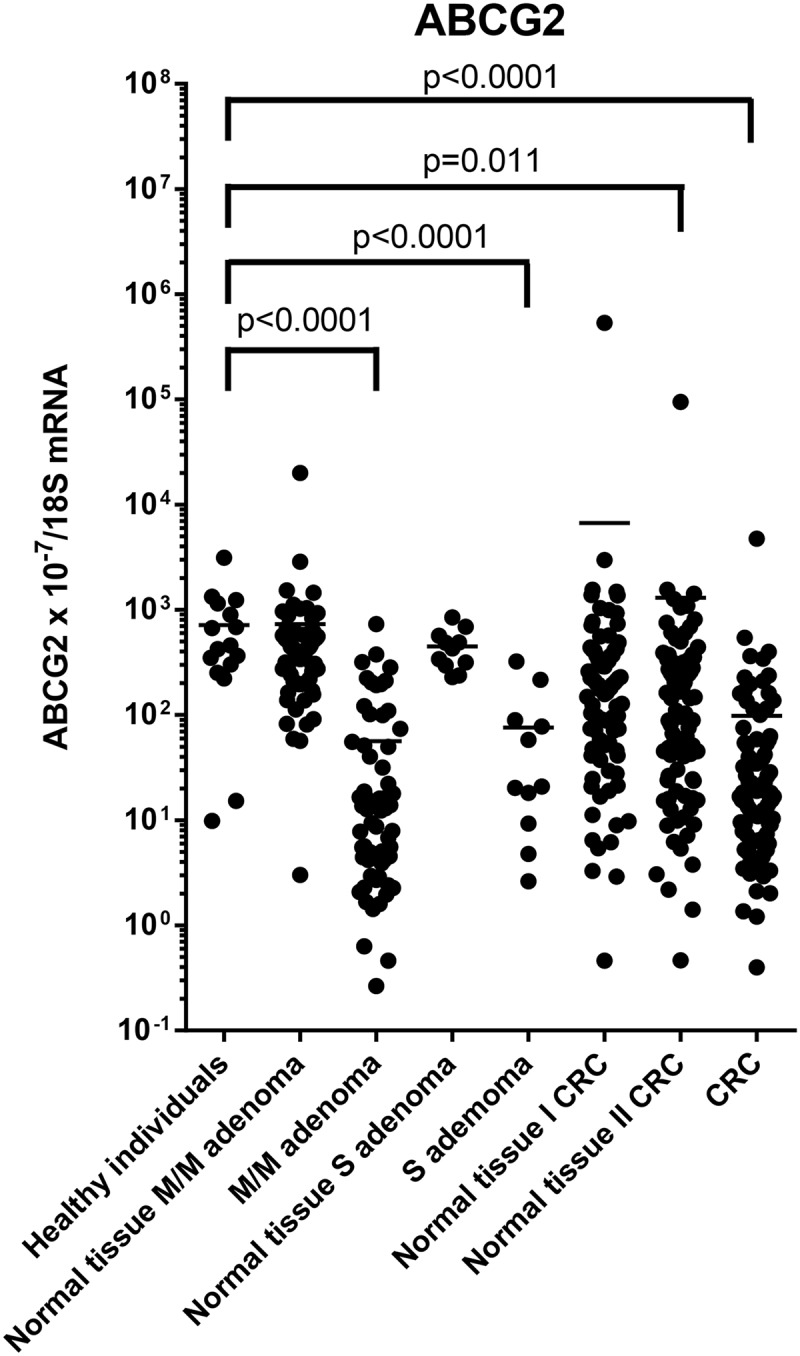
*ABCG2* mRNA levels in morphologically normal and affected tissues normalised to 18S RNA levels. P-values for comparison of the expression levels to the level in healthy controls adjusted for age and gender are indicated. M/M, mild-to moderate dysplasia; S, severe dysplasia; Normal tissue I, morphologically normal distant tissue; Normal tissue II, morphologically normal adjacent tissue. Horizontal bars indicate mean. mRNA expression levels were log-transformed.

*ABCG2* mRNA levels in the intestinal tissue from the healthy individuals, adenoma and CRC cases are shown in Table 3 and Fig. 2.

**Table 3:** *ABCG2* mRNA levels x 10^7^ in intestinal tissues normalised to 18S RNA levels

	unaffected tissue		adenomas/carcinomas		
**Variable**	Mean ± S.D.	P^a^	Mean ± S.D.	P^a^	P^b^
**Healthy individuals**	718.06 ± 761.24				
**Mild moderate dysplasia**	732.85 ± 2305.28	0.55	56.02 ± 118.42	<0.0001	<0.0001
**Severe dysplasia**	448.02 ± 195.34	0.84	76.31 ± 102.63	<0.0001	<0.0001
**Cancer patients**	6679 ± 58353 (distant)	0.080	98.41 ± 476.36	<0.0001	<0.0001
	1302 ± 10090 (adjacent)	0.011			<0.0001

^a^ P-values for comparison of the expression levels in tissue from healthy individuals adjusted for age and gender.

^b^ P value for the comparison of the expression levels in morphologically unaffected and affected tissue from the same individual using Paired Student’s t-test. Matching samples were available from 66 cases with mild-moderate dysplasia, 11 cases with severe dysplasia, and 80 and 83 CRC cases (distant unaffected tissue, and adjacent unaffected tissue, respectively.

*ABCG2* mRNA levels were statistically significantly lower in all adenoma tissues compared to mRNA levels in unaffected tissue from the same individuals and compared to mRNA levels in tissue from healthy individuals (all p<0.0001).

*ABCG2* mRNA were statistically significantly lower in cancer tissue both compared to levels in unaffected tissue from the same person and compared to levels in tissue from healthy controls (all p<0.0001). ABCG2 mRNA levels in unaffected tissue from cancer patients were higher than the level in tissue from healthy individuals, but considerable variation was observed (Fig. 2), and the difference was only statistically significant for adjacent unaffected tissue (p = 0.011).

### Genotype and expression level association

No associations between *NFKB1*–94ins/del (rs28362491) genotypes and *ABCC2* and *ABCG2* mRNA levels were found (S2 Fig).

## Discussion

In the present study we found significantly higher mRNA level of *ABCC2* in adenomas with mild to moderate dysplasia and carcinoma tissue compared to the level in unaffected tissue from the same individuals. The same was found in carcinoma and distant unaffected tissue from CRC cases compared to the level in tissue from healthy individuals. Furthermore, we found significantly lower level of *ABCG2* in adenomas and carcinomas compared to the level in unaffected tissue from the same individuals and as compared to tissue from healthy individuals. The mRNA level of *ABCG2* in adjacent normal tissue was significantly higher than in healthy individuals.

Our findings are in agreement with previous findings in a study of *ABCC2* and *ABCG2* mRNA levels in normal and affected colonic tissue from 51 CRC patients [44], where *ABCC2* mRNA level was higher in tumour tissue compared to unaffected tissue from the same individual, and the *ABCG2* mRNA levels were found to be lower [44]. Our study is also in agreement with a study of ABCG2 and ABCC2 protein and *ABCG2* mRNA levels in 29 adenomas from 21 patients [45]. The authors found low levels of ABCG2 protein and *ABCG2* mRNA in the adenoma tissue using adjacent tissue as control [45]. The latter study found low ABCC2 protein levels in control tissue and no detectable changes in the levels of the adenomas [45]. Moreover, in an animal model, down-regulation of ABCG2 protein expression was found in adenoma tissue compared to adjacent normal tissue [45]. Concurrently, high level of the ABCG2 substrate, the he food carcinogen ation of ABCC2 affe tissue om 21 patients which found that the level of pared to the level infood carcinogen 2-amino-1-methyl-6-phenylimidazo[4,5-β] pyridine (PhIP) and a higher although not statistically significantly increased level of PhIP-DNA adducts were found in the intestinal adenomas as compared to adjacent tissue of these mice [45].

Our study replicates and further corroborates these previous findings by including healthy individuals, and, furthermore, assessed the levels of *ABCC2* and *ABCG2* in the normal-mild dysplasia-severe dysplasia-carcinoma sequence. The finding of lowered *ABCG2* level in adenomas with mild/moderate dysplasia suggests that low ABCG2 activity may hamper export of food carcinogens in adenomas with mild/moderate dysplasia. Thereby, food carcinogens, including PhIP, could accumulate in adenomas and thus promote carcinogenesis. However, we detected interaction between intake of meat and genetic variants in *ABCB1* but not with *ABCG2* [29]. We have previously reported low mRNA levels of *ABCB1* in adenomas and carcinomas and this may suggest similarities in the regulation of gene expression of these two ABC transporters in colorectal carcinogenesis [30].

In the present study, high levels of *ABCC2* gene expression were observed in adenomas with mild/moderate dysplasia similarly suggesting that ABCC2 is involved in the early development of colorectal carcinogenesis. Dietary omega-3 and omega-6 fatty acids are precursors of the arachidonic acid metabolites eicosanoids, which are potent regulators of inflammation [21]. ABCC2 transports LTB_4_ and probably other eicosanoids [26] which are potent stimulators of inflammatory signalling pathways leading to inflammation and cancer. Thus, high ABCC2 activity due to high level of *ABCC2* mRNA could be involved in the translocation of eicosanoids thereby promoting inflammation and carcinogenesis [28].

Regulation of ABC transporter activity may differ in normal and dysplastic/cancer intestinal tissue. Thus, the intestinal *ABCB1* mRNA levels were found to be associated with polymorphisms in *ABCB1* and *NFKB1* (encoding the p50 subunit of the transcription factor nuclear factor κB (NFκB)) in morphologically normal tissue from adenoma case, whereas no association was found in either dysplastic tissue from adenoma cases or in morphologically normal or cancer tissue from CRC cases [30]. Also, various transcription factors involved in xenobiotic sensing, including pregnane X receptor (PXR), seem to be involved in the regulation of *ABCB1*, *ABCC2* and *ABCG2* mRNA levels in the normal intestine [7,46,47]. Inflammation is an indispensable part of colorectal carcinogenesis [4] and inflammation has been found to down-regulate the expression of intestinal ABC transporters in animal studies [47–49]. In line with this, *ABCB1* and *ABCG2* mRNA levels were decreased in inflammatory intestinal tissue from patients with active intestinal inflammation [50]. Also, pro-inflammatory cytokines produced in the course of inflammation [4] modulates the expression of the ABC transporters [51]. The present study found no association between the previously reported *NFKB1*–94ins/del (rs28362491) polymorphism and *ABCC2* and *ABCG2* mRNA levels.

Multidrug resistance caused by high activity of ABC transporters is a frequent problem in the treatment of CRC [52]. The results of the present study suggest that high ABCC2 activity in cancer tissue even before drug treatment could contribute to inherent multidrug resistance, i.e. that the patient does not respond to initial chemotherapy. Other mechanisms such as selection of cancer cells overexpressing ABC transporters and induction of ABC transporters contribute to the phenotypic development of acquired drug resistance, i.e. where initial treatment is effective but the patient subsequently develops drug resistance [52].

Interestingly, genetic variation in these two ABC transporters was not associated with risk of inflammatory bowel disease or CRC in genetic epidemiological studies although suggestive associations between CRC and *ABCG2* rs2622621 and rs1481012 were found [29,53–55]. Also, abcc2 and abcg2 knockout mice seemed to be healthy [56]. In humans, no overt disease has been related to mutations in these transporters [57]. Dubin-Johnson syndrome is caused by several different mutations in ABCC2, and is associated with a failure to excrete conjugated bilirubin glucuronides in the bile, resulting in elevated levels of conjugated bilirubin in the blood [57].

Taken together, the results from the present study suggest that both ABCC2 and ABCG2 are differentially expressed in early carcinogenesis similar to ABCB1. Our results are in accordance with the hypothesis that changes in the ABC transporter activity is not sufficient to initiate cancer development but may increase susceptibility for dietary factors which are substrates of the ABC transporters. Thus, modified transport across the epithelial barrier may represent one of the early events in the adenoma-carcinoma sequence. However, the present study does not add to the knowledge on the underlying biological mechanisms.

In conclusion, this study found that *ABCC2* and *ABCG2* gene expression levels were altered in mild/moderate dysplasia in the normal-adenoma-carcinoma sequence suggesting that these ABC transporters are involved in early carcinogenesis similar to ABCB1.

## Supporting Information

S1 Fig *ABCC2* and *ABCG2* mRNA levels (x10^7^) normalized to either 18S or β-actin. The plot is shown in double log-scale since the variation in mRNA levels spans three orders of magnitude.
(TIF)

S2 Fig Association between *NFKB1*–94ins/del polymorphism and *ABCC2* and *ABCG2* mRNA levels in morphologically normal and affected intestinal tissue from individuals with adenomas (left panel) and carcinomas (right panel). The number of individuals with each genotype is indicated in brackets above the column. At the the bottom, one outlier (extremely high mRNA values) in each panel has been removed for clarity. Normal tissue I, morphologically normal distant tissue; Normal tissue II, morphologically normal adjacent tissue. Horizontal bars indicate mean with standard error. Kruskal-Wallis test was used to compare mean mRNA expression levels followed by Dunn’s post-test.
(TIF)
