# Supplementary figures and images for: High ABCC2 and Low ABCG2 Gene Expression Are Early Events in the Colorectal Adenoma-Carcinoma Sequence
Source: PLoS One. 2015 Mar 20;10(3):e0119255. doi: 10.1371/journal.pone.0119255 (PMC4368545; doi:10.1371/journal.pone.0119255)

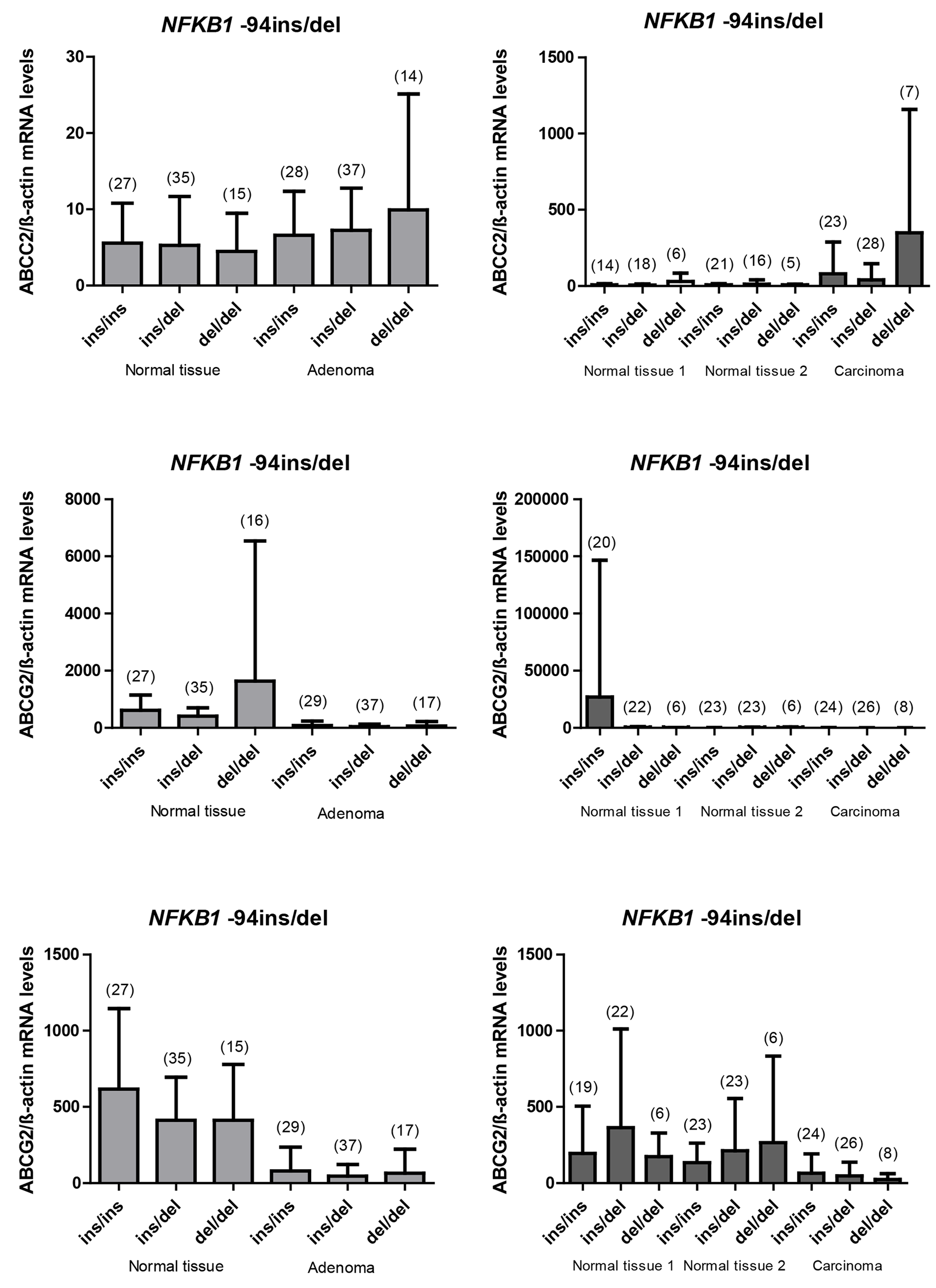

Supplement: S1 Fig — The plot is shown in double log-scale since the variation in mRNA levels spans three orders of magnitude. (TIF) [file pone.0119255.s001.tif]

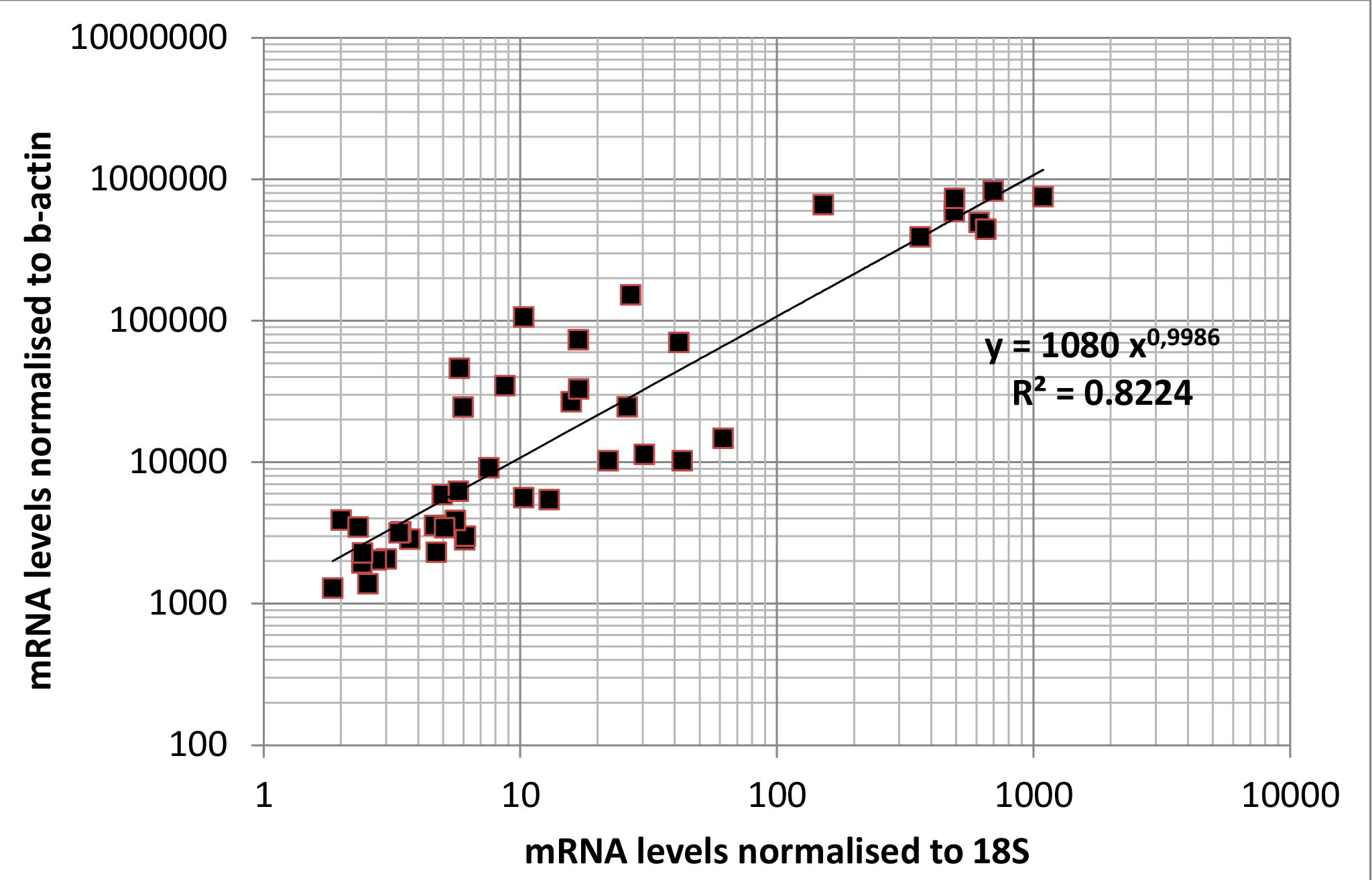

Supplement: S2 Fig — The number of individuals with each genotype is indicated in brackets above the column. At the the bottom, one outlier (extremely high mRNA values) in each panel has been removed for clarity. Normal tissue I, morphologically normal distant tissue; Normal tissue II, morphologically normal adjacent tissue. Horizontal bars indicate mean with standard error. Kruskal-Wallis test was used to compare mean mRNA expression levels followed by Dunn’s post-test. (TIF) [file pone.0119255.s002.tif]
